# Skin Diseases

**Published:** 1892-09

**Authors:** 


### SKIN DISEASES.

When a skin disease breaks out the assumption generally is that the blood is out of order, and that it is full of "humor." What is meant by the term "humor" no one seems to know; yet for generations it has been used, and to-day is quite as popular as ever.

Consonant with this inexplicable theory, the victims of these troubles generally dose themselves with so-called "blood purifiers," until they learn from experience that they were wrong in their assumption, and that the cause is external and acts directly upon the skin itself, or that it exists in some important organ, as the stomach, lungs, kidneys or liver.

Probably there is no class of disease that demands greater skill in diagnosis and treatment than this. In only a few instances, comparatively, is the skin directly at fault. In these the eruptions are generally caused by excessive heat, the bites of insects, or something of the sort. Where such causes are active, and can be determined, a cure can occasionally be effected. But in the majority of eruptions the cause lies beyond the skin, and in some of the important organs mentioned.

In which case it is not easily discovered, even by discerning physicians; and, of course, laymen could rarely ever, if ever, find it.

Considering this fact, self-treatment can promise but little, and really nothing where "blood purifiers" are used. If people will insist upon experimenting upon themselves in this class of affections, at least they should confine themselves to external remedies, and to those that they know to be absolutely harmless. If they wish to use an ointment, let it be the simple oxide of zinc ointment, or one made by adding a teaspoonfull of sulphur to one or two tablespoonfuls of lard. Vaseline is also a very good "salve." Unless the ingredients are carefully selected, and on scientific principles, no other ointment promises better than these. Or if a lotion is preferred, one may be made up of two drachms of oxide of zinc, one ounce of glycerine, and five ounces of rose water. This can do no harm, and it may do much good in some cases. Or if there is much itching and the skin is not broken, it can best be overcome by a solution of menthol in alcohol — one drachm to ten drachms.

As for the indiscriminate use of medicines internally for skin diseases, no good can come from it; moreover, in most cases it must do harm. Internal self-treatment should be restricted to a careful regulation of the diet; and this, in some instances, will do much to effect a cure. Rich dishes such as pastries, gravies, etc., often give rise to indigestion; and this trouble is likely to aggravate, if it does not positively create, a disorder of the skin. On the other hand, contrary to the belief of many people, simple fats, as cream, butter, also fatty parts of meats that have been broiled, roasted or boiled, do not have any injurious effect upon the skin, whether the skin is in a healthy state or not.

In order that medicinal treatment may do good in diseases of the skin without doing harm, it must be specially chosen for every case; that is, in every instance the patient must be carefully studied, and his nature, peculiarities, age, occupation, etc., duly considered. There is no one known remedy that would prove serviceable in any number of cases, for in almost all of them there is something that would call for modifications in treatment. Hence, as stated, in every instance of disorder of the skin that promises to prove obstinate, let a physician be consulted as soon as possible.

Apropos of this, there is an ancient prejudice that in the treatment of skin diseases great care must be used to prevent their "striking in," the idea being that it is possible to drive them to some vital organ. This fear is absolutely groundless ; no harm can possibly come from the disappearance or cure of a skin eruption. So these troublesome and disfiguring affections may safely be speedily cured when possible.

